# Complete mitochondrial genome of *Stonychophora maculata* (Orthoptera: Rhaphidophoridae: Rhaphidophorinae)

**DOI:** 10.1128/mra.01165-25

**Published:** 2026-02-10

**Authors:** Xiang Li, Tingting Yu, Xun Bian, Bin Zhang

**Affiliations:** 1 College of Life Sciences and Technology, Inner Mongolia Normal University 71203 , Hohhot, China; 2 Guangxi Key Laboratory of Rare and Endangered Animal Ecology, Guangxi Normal University 12388, Guilin, China; 3 Key Laboratory of Biodiversity Conservation and Sustainable Utilization for College and University of Inner Mongolia Autonomous Region, Hohhot, China; University of Maryland School of Medicine, Baltimore, Maryland, USA

**Keywords:** mitogenome, Malaysia, Rhaphidophorinae

## Abstract

We present the complete mitochondrial genome of *Stonychophora maculata* from Malaysia, which is a circular DNA molecule with a total length of 15,956 bp and is AT-rich (74%). It contains 37 mitochondrial genes, including 13 protein-coding genes, two rRNA genes, 22 tRNA genes, and a control region.

## ANNOUNCEMENT

The family Rhaphidophoridae is a distinctive group of orthopterans lacking wings, stridulatory organs, and fore-tibial tympana. They are widely distributed from South Asia to Australia (1). *Stonychophora maculata* Gorochov, 2010, was first reported from “Trus Madi” Mt., Sabah State, Northern Kalimantan, Malaysia. Distinguished by spotted pronotum and tegmina, elongate male cerci with apically widened tips, and a subgenital plate with two lateral lobes; male genitalia with a slender, curved epiphallic apical process and paired medial lobes (2). Identification followed Gorochov (2010) using a Leica DFC550 stereomicroscope; a voucher was deposited in the Insect Collection of Guangxi Normal University (GXNU) (HZ2113). To contribute to the phylogenomics of Rhaphidophorinae, the complete mitochondrial genome of *S. maculata* from Malaysia was assembled and annotated.

The specimen of *S. maculata* analyzed here was collected from Trusmadi Range, Sabah, Borneo, Malaysia (5.59533 N 116.51711E). The voucher specimen was preserved in absolute ethanol at −4°C and deposited in the College of Life Sciences, GXNU. Total genomic DNA was isolated from the muscle tissue of the postfemur using the TIANamp Genomic DNA Kit (TIANGEN) according to the manufacturer’s protocol, and subsequently subjected to high-throughput sequencing at Beijing Berry Genomics Co., Ltd. A 150 bp paired-end library was constructed with the MGIEasy Kit (MGI) as well as sequenced on an Illumina NovaSeq 6000 (Illumina Inc.). The raw data were processed with fastp v.0.20.0 (3) by trimming adapters and primers, filtering reads with phred quality <Q5, and filtering reads with N base number >3. The sequencing generated 23,054,251 reads that were filtered. The number of reads listed is after QC. The entire mitochondrial genome sequence was assembled by NOVOPlasty 4.3.3 (4) using type mito, genome range 14,000–18,000, kmer 39, max memory 16, extended log 0, and *Rhaphidophora duxiu* (PP953500) as reference sequence. A single *S. maculata* mitochondrial contig with 191× coverage was identified by Quast 5.2.0 using the default settings (5). Genome annotation was performed employing MITOS 2 (6) on the Galaxy platform (https://usegalaxy.org/). Terminal overlap was examined and trimmed in Geneious Prime 2025.02 (7), with the sequence origin reset to the tRNA-I gene (8). Gene boundaries were verified against 33 complete Rhaphidophoroidea mitochondrial genomes in GenBank using standard invertebrate initiation and termination codons (9, 10). Nucleotide identities were calculated by BLAST 2.15.0+ search using the default settings (11).

**Fig 1:**
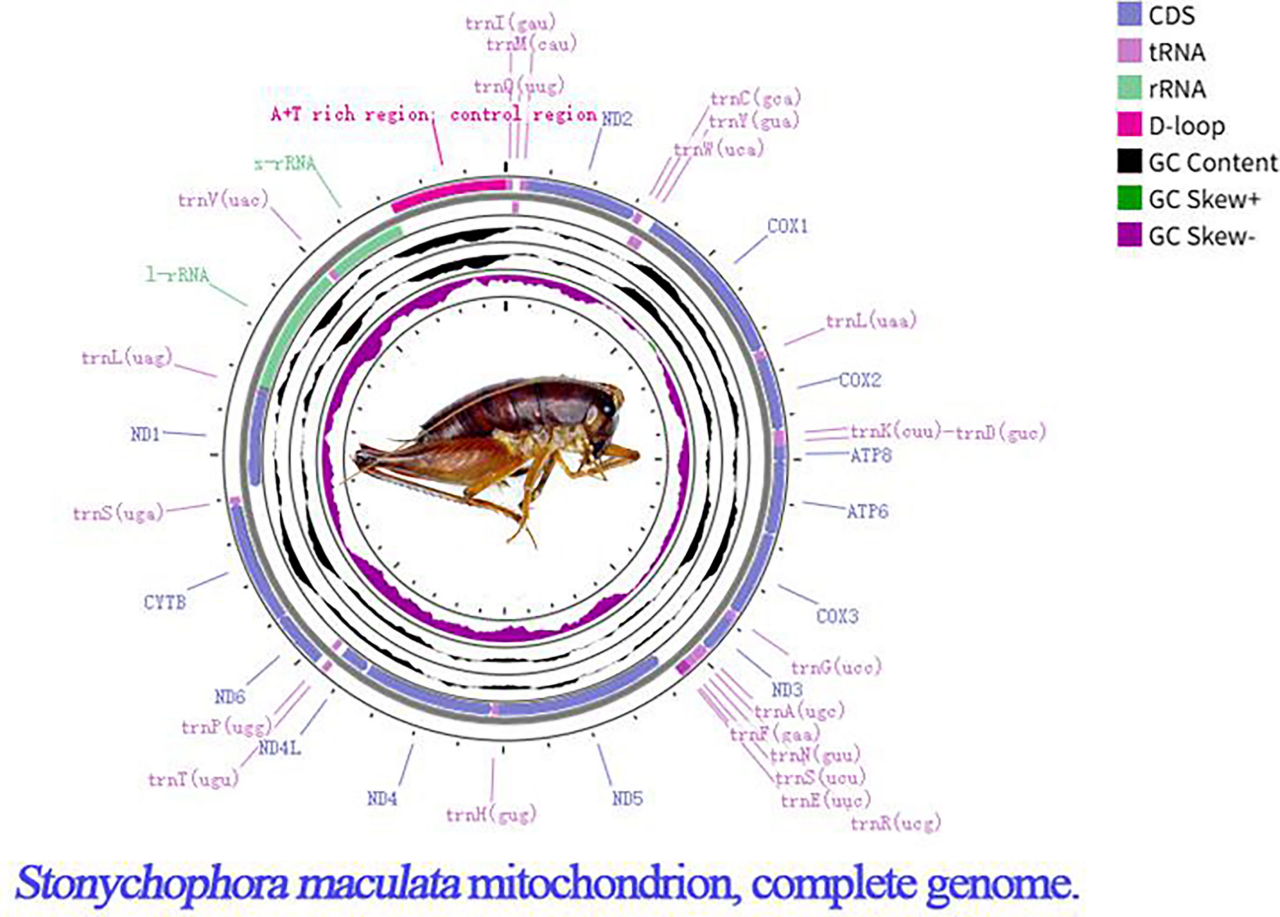
Complete mitochondrial genome map of *Stonychophora maculata*. The mapping was performed with Proksee (https://proksee.ca/) (14). From the inner ring to the outer ring, the genomic map sequentially displays the GC skew, GC content, and its composition, as well as gene positions.

The complete circular mitochondrial genome of *S. maculata* is 15,956 bp in length and has an AT bias of 76% (PX412918). The GC content is 26%. The complete mitogenome contains 37 genes, including 13 protein-coding genes, 2 ribosomal RNA genes, 22 transfer RNA genes, and a control region (D-loop) (Fig. 1). All protein-coding genes initiate with ATN (Table 1). The TAA termination codon is found in all genes, “except COX2, ND4, and ND5; the TAA termination codon is generated by the addition of 3′ A residues to the mRNA, as is common in animal mitochondrial genomes (12, 13).” The entire mitochondrial genome sequence of *S. maculata* is 86.07% similar to the genome of *Rhaphidophora duxiu* from China.

**TABLE 1:** Annotation and gene organization of the *Stonychophora maculata*

Gene	Type	Minimum nucleotide position	Maximum nucleotide position	Length	Start codon	Stop codon	Direction
*tRNA-Ile*	tRNA	1	67	67	–^*a*^	–	Forward
*tRNA-Gln*	tRNA	64	133	70	–	–	Reverse
*tRNA-Met*	tRNA	132	201	70	–	–	Forward
*ND2*	CDS	202	1230	1,029	ATG	TAA	Forward
*tRNA-Trp*	tRNA	1240	1308	69	–	–	Forward
*tRNA-Cys*	tRNA	1300	1364	65	–	–	Reverse
*tRNA-Tyr*	tRNA	1365	1433	69	–	–	Reverse
*COX1*	CDS	1426	2967	1,542	ATT	TAA	Forward
*tRNA-Leu*	tRNA	2974	3040	67	–	–	Forward
*COX2*	CDS	3041	3731	691	ATT	T	Forward
*tRNA-Lys*	tRNA	3731	3801	71	–	–	Forward
*tRNA-Asp*	tRNA	3800	3869	70	–	–	Forward
*ATP8*	CDS	3870	4028	159	ATT	TAA	Forward
*ATP6*	CDS	4022	4699	678	ATG	TAA	Forward
*COX3*	CDS	4703	5491	789	ATG	TAA	Forward
*tRNA-Gly*	tRNA	5498	5565	68	–	–	Forward
*ND3*	CDS	5566	5919	354	ATT	TAA	Forward
*tRNA-Ala*	tRNA	5921	5985	65	–	–	Forward
*tRNA-Arg*	tRNA	5984	6048	65	–	–	Forward
*tRNA-Asn*	tRNA	6052	6119	68	–	–	Forward
*tRNA-Ser*	tRNA	6119	6186	68	–	–	Forward
*tRNA-Glu*	tRNA	6186	6250	65	–	–	Forward
*tRNA-Phe*	tRNA	6250	6323	74	–	–	Reverse
*ND5*	CDS	6320	8051	1,732	ATT	T	Reverse
*tRNA-His*	tRNA	8051	8116	66	–	–	Reverse
*ND4*	CDS	8117	9437	1,321	ATA	T	Reverse
*ND4L*	CDS	9449	9742	294	ATG	TAA	Reverse
*tRNA-Thr*	tRNA	9748	9814	67	–	–	Forward
*tRNA-Pro*	tRNA	9814	9879	66	–	–	Reverse
*ND6*	CDS	9881	10408	528	ATT	TAA	Forward
*CYTB*	CDS	10408	11544	1,137	ATG	TAA	Forward
*tRNA-Ser*	tRNA	11543	11612	70	–	–	Forward
*ND1*	CDS	11685	12629	972	ATA	TAA	Reverse
*tRNA-Leu*	tRNA	12635	12701	67	–	–	Reverse
*16SrRNA*	rRNA	12695	13981	1,287	–	–	Reverse
*tRNA-Val*	tRNA	14011	14083	73	–	–	Reverse
*12SRNA*	rRNA	14083	14875	793	–	–	Reverse

^*a*^ –, not applicable.

## Data Availability

The complete mitochondrial genome sequence of *Stonychophora maculata* is available in GenBank under accession number PX412918. The associated BioProject, SRA, and BioSample numbers are PRJNA1335790, SRR35974945, and SAMN52020757, respectively. The mitochondrial genome referenced in the text is *Rhaphidophora duxiu* GenBank accession number PP953500.
